# Supplementary material for: Graphene Oxide/Polyvinyl Alcohol–Formaldehyde Composite Loaded by Pb Ions: Structure and Electrochemical Performance
Source: Polymers (Basel). 2022 Jun 6;14(11):2303. doi: 10.3390/polym14112303 (PMC9183114; doi:10.3390/polym14112303)
Supplement: Supplementary file 1 [file polymers-14-02303-s001.zip › polymers-1752846-supplementary.pdf]

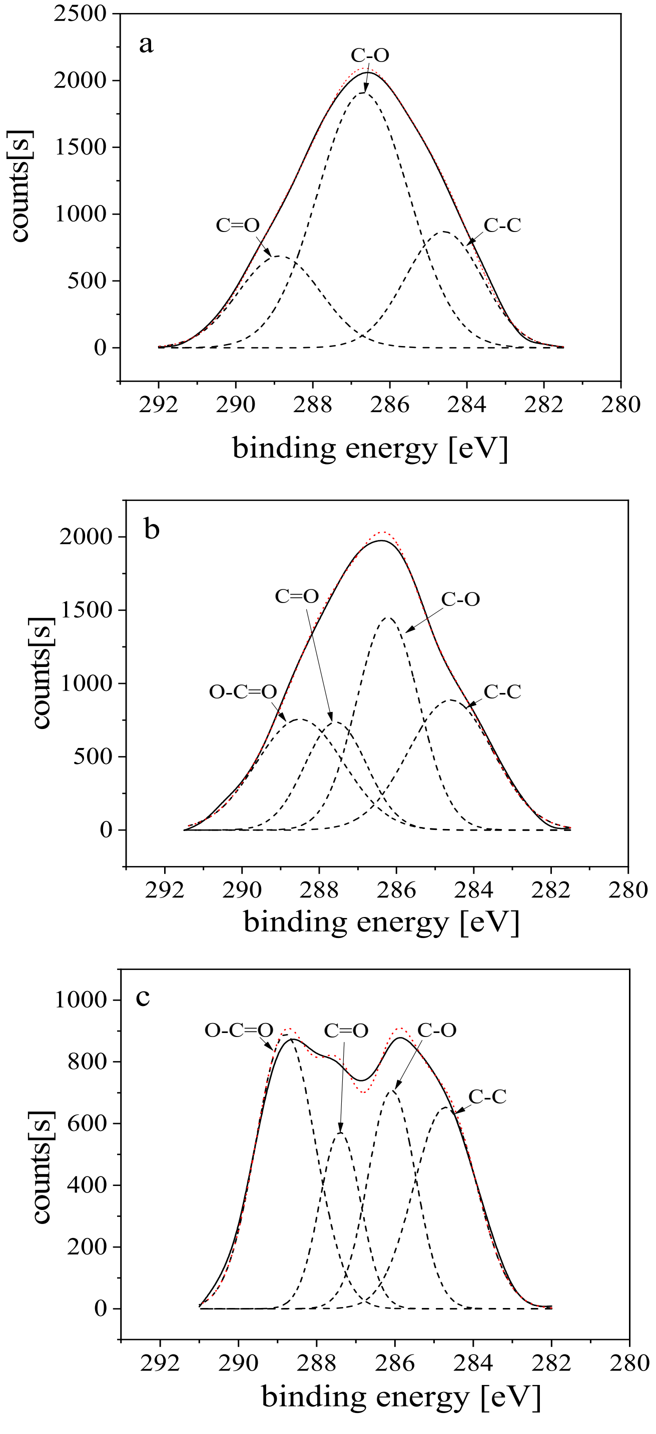

Figure S1. High-resolution XPS spectra of the C1s peak of (a) the blank PVF polymer, (b) the PVF/GO and (c) the PVF/GO/Pb composite. The solid line represents the experimental data where the dashed lines represent the individual contributions. The dotted red line is a fit of a sum of three or four Gaussians to the data.

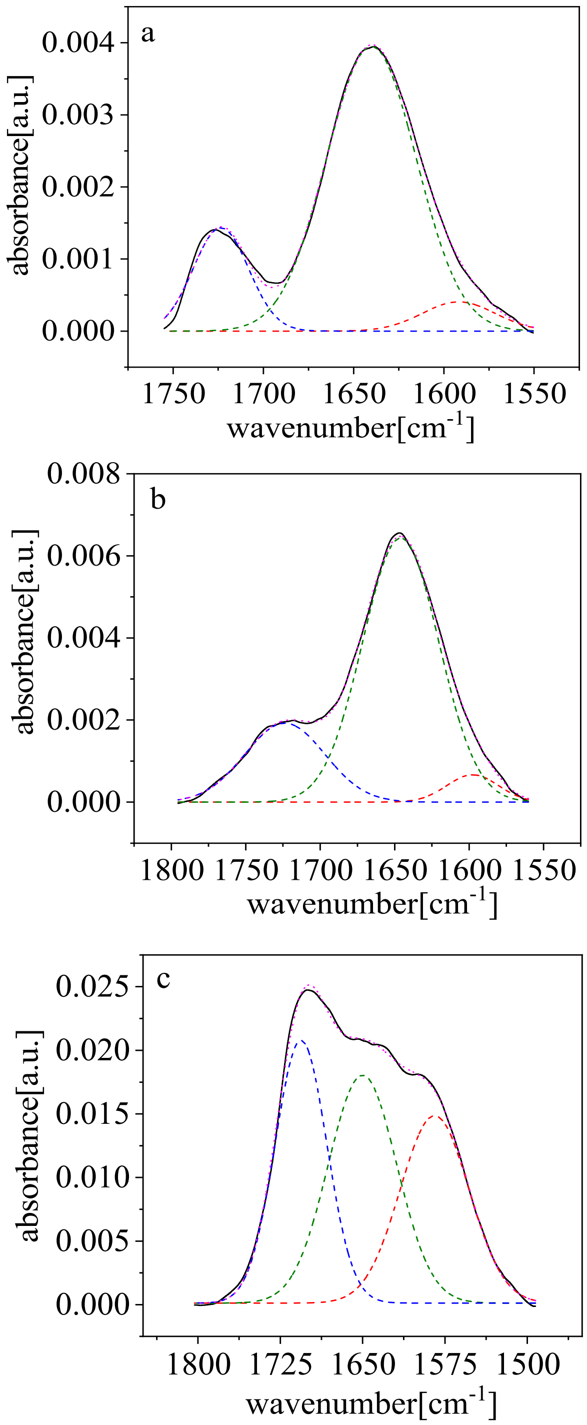

Figure S2. FTIR spectra of stretching band from 1500 to 1800 cm^{-1} for the different materials de-convoluted into three peaks (a) PVF, (b) PVF/GO and (c) PVF/GO/Pb composite.

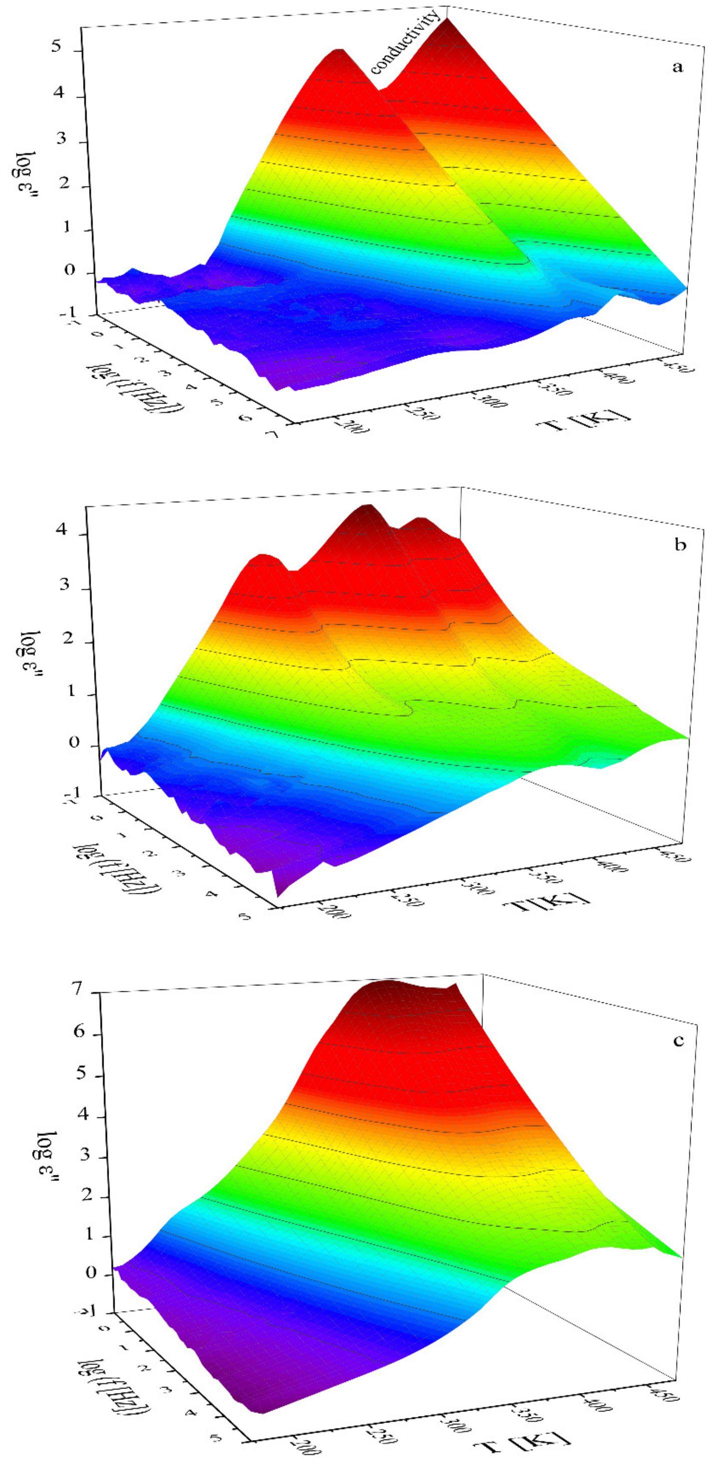

Figure S3. 3D representation of the dielectric loss (log ε'') as function of frequency and temperature for the heating cycle of (a) PVF, and (b) PVF/GO and (c) PVF/GO/Pb composites.

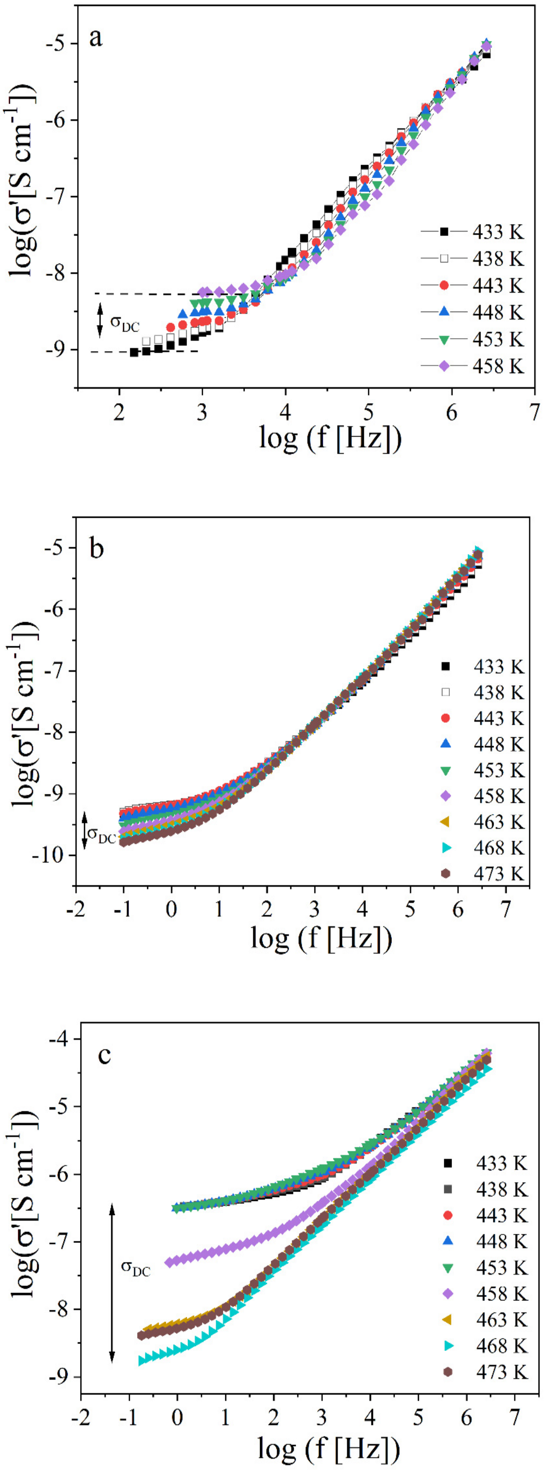

Figure S4. Real part of the complex conductivity σ' vs. frequency f at the indicated temperatures on the heating run; (a) PVF, (b) PVF/GO and (c) PVF/GO/Pb.
